# Seed Germination Ecology of the Cold Desert Annual *Isatis violascens* (Brassicaceae): Two Levels of Physiological Dormancy and Role of the Pericarp

**DOI:** 10.1371/journal.pone.0140983

**Published:** 2015-10-29

**Authors:** Yuan M. Zhou, Juan J. Lu, Dun Y. Tan, Carol C. Baskin, Jerry M. Baskin

**Affiliations:** 1 Xinjiang Key Laboratory of Soil and Plant Ecological Processes, College of Grassland and Environment Sciences, Xinjiang Agricultural University, Urümqi, China; 2 Department of Biology, University of Kentucky, Lexington, Kentucky, United States of America; 3 Department of Plant and Soil Sciences, University of Kentucky, Lexington, Kentucky, United States of America; Kunming Institute of Botany, CHINA

## Abstract

The occurrence of various species of Brassicaceae with indehiscent fruits in the cold deserts of NW China suggests that there are adaptive advantages of this trait. We hypothesized that the pericarp of the single-seeded silicles of *Isatis violascens* restricts embryo expansion and thus prevents germination for 1 or more years. Thus, our aim was to investigate the role of the pericarp in seed dormancy and germination of this species. The effects of afterripening, treatment with gibberellic acid (GA_3_) and cold stratification on seed dormancy-break were tested using intact silicles and isolated seeds, and germination phenology was monitored in an experimental garden. The pericarp has a role in mechanically inhibiting germination of fresh seeds and promotes germination of nondormant seeds, but it does not facilitate formation of a persistent seed bank. Seeds in silicles in watered soil began to germinate earlier in autumn and germinated to higher percentages than isolated seeds. Sixty-two percent of seeds in the buried silicles germinated by the end of the first spring, and only 3% remained nongerminated and viable. Twenty to twenty-five percent of the seeds have nondeep physiological dormancy (PD) and 75–80% intermediate PD. Seeds with nondeep PD afterripen in summer and germinate inside the silicles in autumn if the soil is moist. Afterripening during summer significantly decreased the amount of cold stratification required to break intermediate PD. The presence of both nondeep and intermediate PD in the seed cohort may be a bet-hedging strategy.

## Introduction

In the context of gaining a better understanding of seed germination of desert species whose diaspores are dry indehiscent fruits, we have focused on the cold deserts of central Asia, in particular the Gurbantunggut Desert in the Junggar Basin of Xinjiang Uyghur Autonomous Region of northwest China. In this desert, there are at least eight genera and 31 species of annual ephemerals in five tribes of Brassicaceae, 11 of which produce dry indehiscent silicles or siliques [1,2]. The occurrence of various Brassicaceae species with indehiscent fruits suggests that there are some adaptive advantages of this trait in arid zones with unpredictable rainfall, such as the Gurbantunggut Desert. But what are they? One possibility is that the pericarp plays an important role in delaying seed germination, which may be a hedge against the risk associated with germination in a temporally unpredictable environment [3,4].

According to the Nikolaeva-Baskin classification system, there are five classes of seed dormancy [5,6]. Physiological dormancy is caused by low growth potential of the embryo, physical dormancy by a water-impermeable seed or fruit coat, combinational dormancy by a water-impermeable seed (or fruit) coat and low growth potential of the embryo, morphological dormancy by an underdeveloped embryo that needs to complete growth (the dormancy period) within the mature seed before the radicle emerges (i.e. seed germinates) and morphophysiological dormancy by an underdeveloped embryo that also is physiologically dormant. Of these, the only one known for Brassicaceae species is physiological dormancy (PD). PD occurs in three increasing degrees or depths (intensities) of dormancy as follows: nondeep PD < intermediate PD < deep PD.

In fresh (nontreated) seeds with nondeep PD and intermediate PD, isolated embryos give rise to normal seedlings, although compared to embryos from treated (nondormant) seeds there may be a bit of a lag in time in beginning of growth of the embryo into a seedling. In constrast, embryos isolated from fresh (nontreated) seeds with deep PD either do not grow or if they do the seedling is abnormal [7]. Nondeep PD is broken in seeds of many species by 2–8 weeks of warm stratification (or sometimes by 8–12 weeks of afterripening in dry storage), but it is broken in seeds of other species by 2–10 weeks of cold stratification [6,7]. Seeds of temperate/arctic-zone species with intermediate or deep PD require a minimum of 4–24 and 8–25 weeks, respectively, of cold stratification, depending on species, for dormancy to be broken [6]. However, a pretreatment period of afterripening or of warm stratification may reduce the length of the cold stratification period required to break intermediate PD [7]. Gibberellic acid (GA_3_) will break nondeep PD, may or may not break intermediate PD, depending on the species, and does not break deep PD [7].

Previous research has shown that the pericarp can prevent germination via mechanical restriction [8–11], chemical inhibition [12,13] or both mechanical restriction and chemical inhibition [14–16], all of which are components of PD [5]. This diversity of mechanisms indicates that the effect of the pericarp on dormancy and germination is complex. However, with a few exceptions [10,16] little information on the role of the pericarp in seed dormancy/germination is available for cold desert species of Brassicaceae. In *Diptychocarpus strictus*, one dispersal morph is a winged and mucilaginous seed, and the other is an indehiscent silique [10]. In *Lachnoloma lehmannii*, the silicles are indehiscent and thickly-covered with long trichomes [16]. In both of these Brassicaceae species, the pericarp strongly inhibits seed germination. After 2 years of burial in soil, no seeds of *L*. *lehmannii* inside the pericarp (silicles) had germinated, although they were viable [16]. Seeds inside siliques of *D*. *strictus* did not begin to germinate until they had been buried for 14 months, and some germinated in the fifth autumn after burial [17]. To increase our knowledge of the germination ecology of cold desert Brassicaceae, we investigated seed dormancy/germination of *Isatis violascens* Bunge, whose silicles are indehiscent.

**Fig 1:**
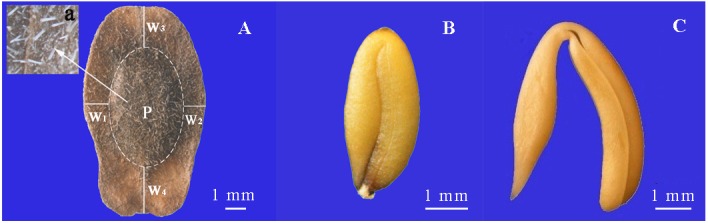
Morphology of silicle (A), seed (B) and embryo (C) of *Isatis violascens*. a (inset), trichomes. W_1_, W_2_, W_3_ and W_4_, wing width. W_1_ is left side of silicle; W_2_ right side of silicle; W_3_ upper end of silicle; and W_4_ peduncle end of silicle. P, seed covered by pericarp.

*Isatis violascens* is an annual ephemeral that occurs in central Asia. In China, the species is a component of the spring ephemeral flora of fixed sand surfaces in the interidge zone and middle-lower slopes of sand dunes in the southern part of the Garbantunggut Desert in Xinjiang [18–20]. It has been suggested that this species be considered as a synonym of *I*. *emarginata* [21]. Ma and Tan [22] reported that *I*. *violascens* germinates in late March, fruits are fully mature in late May and early June and the length of the life cycle is about 70 d. Each silicle has a wing and contains one seed (Fig 1). Based on results from other studies of indehiscent fruits of cold desert Brassicaceae [10,16], we hypothesized that the silicle pericarp of *I*. *violascens* restricts embryo expansion and thus prevents germination until the second (or some later) year after maturation, thereby allowing time for the pericarp to soften and release mechanical and/or chemical restriction of germination. To test this hypothesis, we compared the dormancy-breaking effects of (1) afterripening, (2) the plant growth regulator gibberellic acid (GA_3_) and (3) cold stratification (see [6]) on seeds in intact silicles and isolated seeds in the laboratory and (4) monitored their germination phenology in an experimental garden.

Annual species that grow in unpredictable habitats and flower in spring and set seeds before the onset of summer drought may behave as obligate winter annuals or as facultative winter annuals. Seeds of obligate winter annuals germinate only in autumn, whereas those of facultative winter annuals can germinate in autumn and in spring. Plant from autumn-germinating seeds behave as winter annuals, whereas those from spring-germinating seeds behave as spring ephemerals [6]. The series of experiments designed to test the hypothesis that the pericarp of *I*. *violascens* delays germination also provides insight into how the seed stage of this species differs from that of other facultative winter annuals studied thus far.

## Material and Methods

### Ethics approval

No specific permits were required for the described field studies. The location is not privately-owned or protected in any way, and the field studies did not involve endangered or protected species.

### Field site description and silicle collection

The field study site is a cold desert sand dune in Fukang city in the southern part of the Junggar Basin of Xinjiang Province (44°22′N, 88°08′E, 458 m a.s.l.), China. This area of the Junggar Basin has a temperate continental climate. Mean annual temperature is 7.9°C, and the mean temperature of the coldest (January) and hottest (July) months is -17.0°C and 26.0°C, respectively. Average annual precipitation (including rain and snow) is 202.2 mm, about two-thirds of which falls in spring and summer. The snow that falls in winter begins to melt in March or April (data from Fukang weather station, 2001–2013). Annual potential evaporation is > 2000 mm [23].

Freshly-matured silicles were collected on 8 June 2013 from dry infructescences of *I*. *violascens* plants growing in three natural populations, each consisting of several hundred plants. The number of silicles ranged from 15–200 per individual, depending on plant size. The silicles from the three populations were pooled and stored in paper bags at room conditions (16–30°C, 10–40% RH) until used.

### Morphological characteristics of silicles and seeds

Color, shape, size and mass were determined for silicles and seeds that had been stored in laboratory conditions for about 2 mo (Fig 1). Such information would be useful for researchers comparing the seed biology of *I*. *violascens* with that of other species. Length and width of silicles, thickness of pericarp and width of wings were measured for 30 silicles using digital calipers. Seeds were removed from silicles using a razor blade, being careful not to damage the seed coat or embryo. The length and width of seeds and morphology and color of embryos were determined and recorded. In addition, four replications of 100 intact silicles were weighed individually using a Sartorius BS210S electronic-balance (0.0001 g), after which the pericarps and seeds for each replication were weighed separately to determine the relative mass of both components of the dispersal unit.

### Germination ecophysiology

#### Effect of pericarp on imbibition and dehydration of seeds

To determine the amount and rate of water uptake, each of 270 2-mo-old intact silicles and each of 15 isolated seeds were weighted (time 0) and placed in individual 9-cm-diameter Petri dishes on filter paper moistened with distilled water and kept on a laboratory bench at room conditions. After 0.5 h and then at 1-h intervals (until mass was constant), 15 silicles and 15 isolated seeds was removed from the dishes, blotted dry with filter paper and weighed individually. After weighing, isolated seeds were returned to the dishes, and the 15 silicles were cut open and their seeds removed and weighed.

Dehydration of fully imbibed silicles/seeds was monitored. Ninety silicles and 15 isolated seeds that were fully imbibed were weighted (time 0) and placed on dry filter paper at laboratory conditions. After 0.5 h and then at 1-h intervals (until mass was constant), each of 15 silicles and each of 15 isolated seeds was removed from its dish and weighed. Also, at each time interval 15 silicles were weighed, cut open and their seeds removed and weighed.

A one-way ANOVA was used to determine significant differences (p = 0.05) in percentages of increase in mass after imbibition among the three treatments. Tukey’s HSD test was performed for multiple comparisons to determine significant differences (p = 0.05) among the treatments. All data analyses were performed with SPSS version 16.0 (SPSS Inc., Chicago, Illinois, USA) [24].

#### Effect of dry storage (afterripening) on germination

To determine if dormancy-break occurs during dry storage (afterripening), silicles and isolated seeds stored in laboratory conditions (16–30°C, 10–40% RH) for 0 (fresh), 1, 2, 3, 6, 9 and 12 mo were tested for germination. Silicles and seeds were incubated at daily (12/12 h) temperature regimes of 5/2, 15/2 and 30/15°C in light (12 h of ≈ 100 μmol m^-2^ s^-1^, 400–700 nm, cool white fluorescent light each day) or in constant dark (Petri dishes with seeds in them placed in light-proof black bags) for 28 d. For each combination of treatments [seven storage periods × three temperature regimes × two light treatments × two pericarp treatments (i.e. silicles and isolated seeds)], four replicates of 25 silicles or of 25 seeds were incubated on two layers of Whatman No.1 filter paper moistened with 2.5 mL of distilled water in 9-cm-diameter Petri dishes. The 5/2°C regime represents late and early winter, 15/2°C spring and autumn and 30/15°C summer [25]. A seed was considered to be germinated when the radicle had emerged. Germination in light was examined daily for 28 d; germinated seeds were removed at each counting. Seeds incubated in dark were checked only after 28 d; therefore, they were not exposed to any light during the incubation period.

After the germination trials were complete, the nongerminated seeds were tested for viability. Seeds were cut open and the embryo observed. Seeds with white, firm embryos were counted as viable, and those with tan, soft embryos were considered nonviable and excluded from the calculations of germination percentages. Only a very few seeds were nonviable. The tests of fresh seeds (0 mo old) were initiated on 13 June 2013, using seeds collected on 8 June 2013.

Germination data were analyzed using generalized linear models (GLMs). A binomial linear model was fitted with a logit link to germination as the response variable. The models included four fixed effects, i.e. light, storage time, temperature and treatment (intact silicles and isolated seeds) and the interactions between treatment and the other effects (i.e. light, storage time and temperature). Tukey’s HSD test was performed for multiple comparisons to determine significant differences (p = 0.05) within silicles and within seeds in final germination percentages among light conditions and storage time.

#### Effect of GA3 on dormancy break

To test the effects of gibberellic acid (GA_3_) on dormancy break of silicles and of isolated seeds, four replicates of 25 silicles and of 25 isolated seeds were incubated in 0 (distilled water control), 0.1, 1.0 and 10 mmol L^-1^ GA_3_ solutions at daily temperature regimes of 5/2 and 15/2°C in light and in constant dark for 28 d. This experiment was conducted on fresh isolated seeds and intact silicles and on those that had been stored dry at room conditions for 1 and 12 months.

A one-way ANOVA was used to determine significant differences (p = 0.05) in germination percentages among light conditions and GA_3_ treatments of seeds within silicles and of isolated seeds stored for 0, 1 and 12 months. Tukey’s HSD test was performed for multiple comparisons to determine significant differences (p = 0.05).

#### Effect of cold stratification on germination of seeds inside silicles

The purpose of this experiment was to determine if cold stratification is required to break seed dormancy. Four replicates each of 25 0- and 6-mo-old dry-stored silicles were cold stratified on moist filter paper at 4°C in constant dark for 0, 4, 8 and 12 weeks. After each cold stratification period, silicles were incubated in light at 5/2 and 15/2°C for 28 d.

Final germination percentages between the two temperature regimes (i.e. 5/2 and 15/2°C) of 0- and 6-mo-old dry-stored silicles cold stratified for 0, 4, 8 and 12 weeks were compared with the chi-square test to determine significant differences (p = 0.05). A one-way ANOVA was used to determine significant differences (p = 0.05) within 5/2°C and within 15/2°C in final germination percentages of 0- and of 6-mo-old dry-stored silicles among cold stratification periods. Tukey’s HSD test was performed for multiple comparisons to determine significant differences (p = 0.05).

#### Effect of storage in soil in the field on germination and viability

The purpose of this experiment was to determine when silicles and isolated seeds germinate under field (experimental garden) conditions. Five days after collection on 8 June 2013, 200 silicles were placed in each of 20 fine-mesh nylon bags, and 200 isolated seeds were placed in each of another 20 bags. Each bag with silicles and each with seeds (with silicles and seeds in a single layer) was buried at a depth of 0.5 cm in soil in plastic pots (23 cm deep and 27.5 cm diameter with drainage holes at the bottom) filled with a mixture of 50% grey desert soil and 50% desert sand. The pots were placed on the soil surface in the experimental garden on the campus of Xinjiang Agricultural University in Urümqi, near the southern edge of the Junggar Basin. Seeds were subjected to natural temperature and soil moisture conditions; temperature data were recorded by an I-button DS1923 buried at a depth of 0.5 cm in a pot of soil.

Except for months with a snow cover on the ground (December 2013 to February 2014), one pot each of 200 buried silicles and of 200 isolated seeds was haphazardly selected and taken to the laboratory at monthly intervals, starting on 19 July 2013 (seeds and silicles buried for 1 mo) and ending on 9 July 2014 (12 months). For each pot of 200 silicles and of 200 isolated seeds, the percentage of in situ germination, dead seeds and viable seeds was determined. Nongerminated firm seeds were tested for germination. For each combination of treatments [retrieval eight times × two pericarp treatments (i.e. silicles and isolated seeds)], four replicates of 25 silicles and of 25 seeds were placed in 9-cm-diameter plastic Petri dishes on two layers of Whatman No.1 filter paper moistened with distilled water and incubated in light at 15/2°C for 28 days. Seeds were examined for germination daily for 28 d, and germinated silicles and seeds were removed at each counting. After the germination trials were complete, the nongerminated seeds were tested for viability, as previously described.

Data on seed fates during burial in the field and during incubation in light at 15/2°C in Petri dishes were analyzed using generalized linear models (GLMs). A multinomial linear model was fitted with a logit link to seed fates of the two stages. For seed burial in the field, the response variable included three categories (germinated during burial, nongerminated but viable seeds and dead seeds). The models included two fixed effects, i.e. retrieval time and treatment (intact silicles and isolated seeds) and their interaction. For seed germination during incubation in light at 15/2°C in Petri dishes, the response variable also included three categories (germinated during incubation, viable seeds and dead seeds). The models included two fixed effects, i.e. retrieval time and treatment (intact silicles and isolated seeds) and their interaction. Tukey’s HSD test was performed for multiple comparisons to determine significant differences (p = 0.05) within silicles and within seeds in percentages of germinated, viable and dead seeds among retrieval times.

#### Germination phenology

The purpose of this experiment was to determine the effect of soil moisture on germination of seeds in silicles and of those removed from silicles under natural temperature conditions. On 18 July 2013, 50 seeds collected on 8 June 2013 were sown at a depth of 0.5 cm in eight plastic pots (23 cm deep and 27.5 cm diameter) filled with soil. Also, on this date 50 silicles were sown at a depth of 0.5 cm in each of eight pots. All pots were placed on the soil surface in the experimental garden, as previously described. Four pots of silicles and four of seeds were watered, and the others were not watered. In the watered treatment, the soil was watered to field capacity every 3 days throughout the experiment, except during the winter, when the soil was frozen, while in the nonwatered treatment the soil received water only via rainfall or snowmelt. Germination (seedlings) was monitored at 7-day intervals from 18 July 2013 to 9 May 2014. Temperature was recorded by an I-button DS1923 buried in soil at a depth of 0.5 cm in one of the pots in which seeds were buried.

A one-way ANOVA was used to determine significant differences (p = 0.05) in final germination percentages among treatments. Tukey’s HSD test was performed for multiple comparisons to determine significant differences (p = 0.05).

## Results

### Morphological characteristics of silicles and seeds

Silicles are pandurate, compressed (Fig 1A), 6.2 ± 0.1 mm (mean ± 1 s.e.) in length and 3.2 ± 0.1 mm in width. Mass of 100 silicles is 0.88 ± 0.02 g. The pericarp is yellow or brown with dense, short-unbranched trichomes (Fig 1A). The membranous wing is 0.1 ± 0.0 mm thick. Wing width at the four locations shown in Fig 1A is 1.4 ± 0.0, 1.5 ± 0.0, 2.3± 0.1 and 3.1± 0.1 mm for W_1_ (on left side of silicles), W_2_ (on right side), W_3_ (on upper end) and W_4_ (on lower (peduncle) end), respectively. Mass of pericarp is 53.3 ± 0.3% of that of the silicle. Seeds are yellow-green, oval, compressed (Fig 1B), 4.3 ± 0.1 mm in length and 1.8 ± 0.0 mm in width. Mass of 100 seeds is 0.41 ± 0.01 g. Seed mass is 46.7 ± 0.3% of that of the silicle. The cotyledons are incumbent (Fig 1C). Mass of 100 embryos is 0.32 ± 0.01 g.

### Germination ecophysiology

#### Effect of pericarp on imbibition and dehydration of seeds

**Fig 2:**
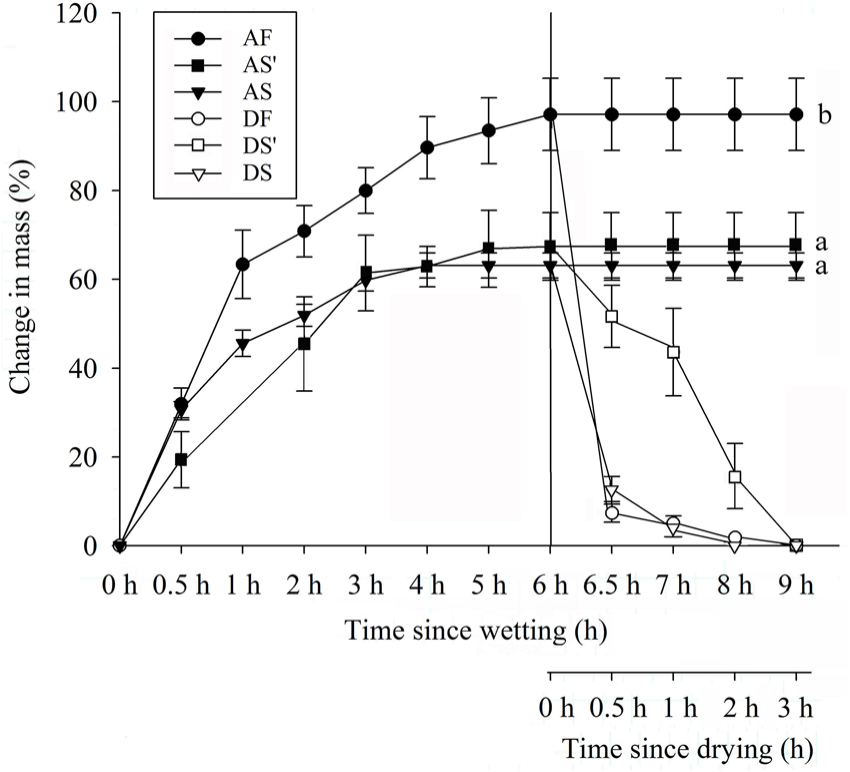
Time course for absorption and dehydration of silicles, seeds inside silicles and isolated seeds of *Isatis violascens*. AF, water absorption by silicles; AS', water absorption by seeds inside silicles; AS, water absorption by isolated seeds; DF, dehydration of silicles; DS', dehydration of seeds inside silicles; DS, dehydration of isolated seeds. Different letters indicate significant differences in final increase in mass. Error bars are ± 1 s.e.

Silicles, seeds inside silicles and isolated seeds imbibed water rapidly during the first 3 h, and they were fully imbibed after 6, 5 and 4 h, respectively (Fig 2). Silicles had a high capacity to take up water, and mass increased 97% in 6 h. After 9 h of imbibition, there were no significant differences in mass of water imbibed by seeds inside silicles and isolated seeds (p = 0.71). Isolated seeds were fully imbibed sooner than those inside the silicles. Silicles, seeds inside silicles and isolated seeds returned to their initial mass after 3, 3 and 2 h of drying, respectively. During dehydration, seeds inside silicles lost water more slowly than isolated seeds.

#### Effect of dry storage (afterripening) on germination

**Fig 3:**
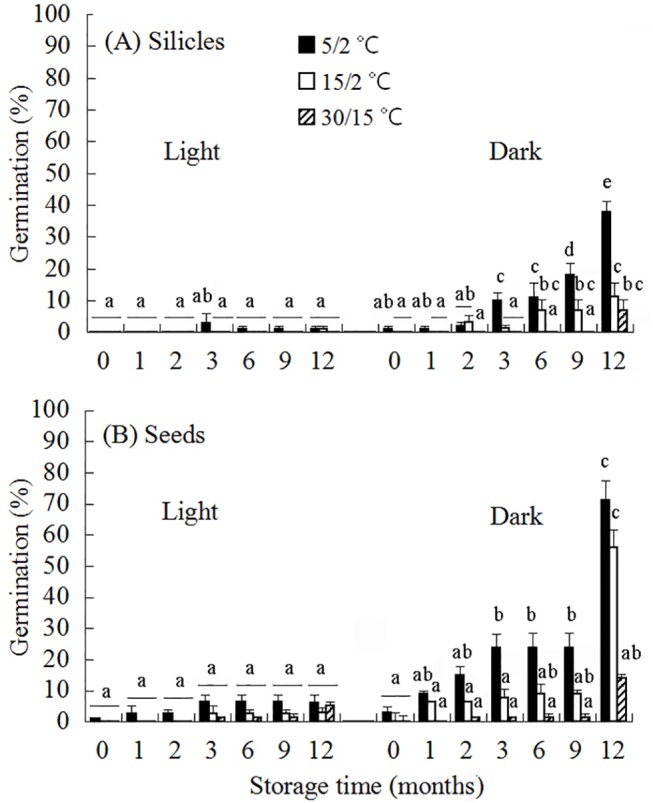
Final germination percentages of seeds in silicles (A) and isolated seeds (B) of *Isatis violascens* incubated in light and in constant darkness at three temperature regimes following 0, 1, 2, 3, 6, 9 and 12 mo of dry storage under laboratory conditions. Bars with different letters are significantly different in multiple range comparison of seeds in silicles and of isolated seeds. Error bars are + 1 s.e.

Analysis of germination during dry storage by GLMs with binomial model (two categories: germinated vs. non-germinated) revealed significant effects of light (χ^2^ = 146.1, p < 0.001), storage time (χ^2^ = 271.9, p < 0.001), temperature (χ^2^ = 153.3, p < 0.001), treatment (χ^2^ = 25.0, p < 0.001) and interaction between storage time and treatment (χ^2^ = 16.3, p = 0.012). However, there was no significant effects of the interaction between temperature and treatment (χ^2^ = 1.1, p = 0.58) or between light and treatment (χ^2^ = 3.2, p = 0.07). At storage time zero, the highest germination was 1% and 3% for seeds in silicles and isolated seeds, respectively. After 6 mo dry storage, the optimum conditions for germination were darkness and 5/2°C, where 11% and 24% of seeds in silicles and isolated seeds germinated, respectively (Fig 3). Additional afterripening occurred between 6 and 12 mo of dry storage, and after 12 mo highest germination of seeds in silicles and isolated seeds was 38% and 71%, respectively.

#### Effect of GA3 on dormancy break

**Fig 4:**
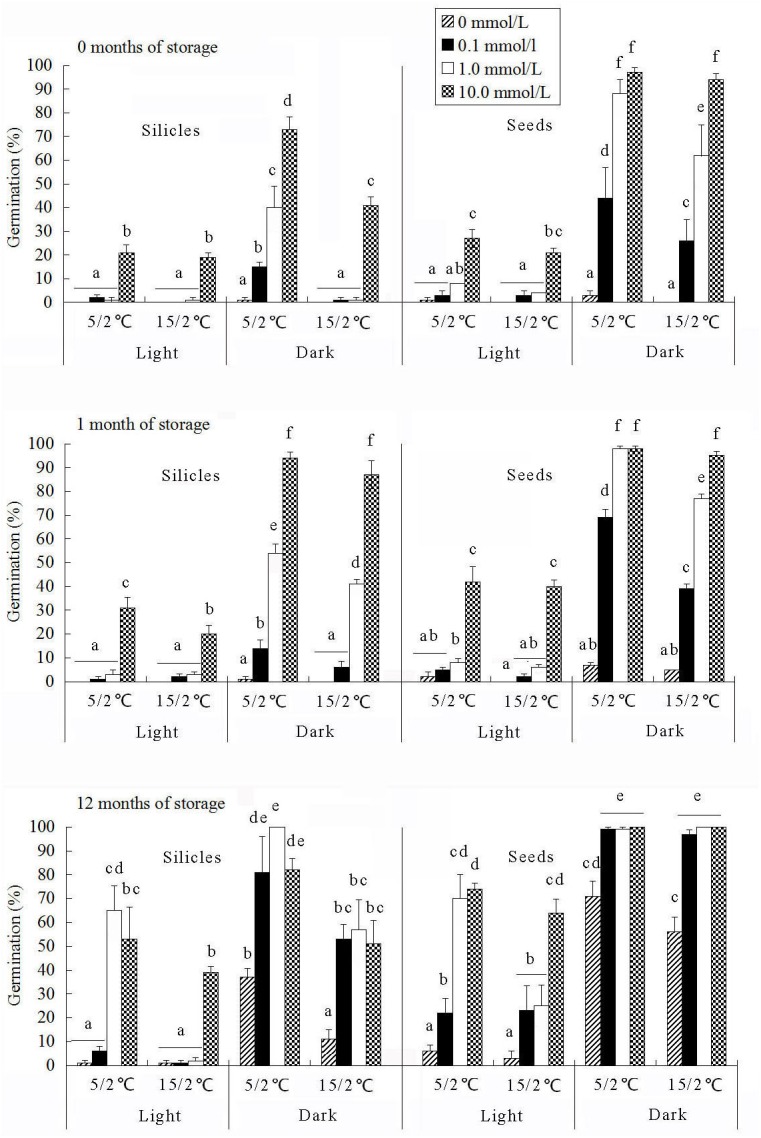
Effect of GA_3_ on germination of seeds in silicles and of isolated seeds of *Isatis violascens* stored dry for 0, 1 and 12 mo and incubated at 5/2 and 15/2°C in light and in constant darkness. Bars with different lowercase letters for silicles and for seeds indicate significant differences in multiple range comparison among incubation conditions. Error bars are + 1 s.e.

Treatment with GA_3_ was a very effective way to break seed dormancy. The highest germination for fresh seeds in silicles was 73%, in dark at 5/2°C in 10 mmol L^-1^ GA_3_, and the highest germination of fresh isolated seeds was 97%, in dark at 5/2°C in 10 mmol L^-1^ GA_3_ (Fig 4). With an increase in seed age, germination in light and in darkness in 1 and 10 mmol L^-1^ GA_3_ and in darkness in 0.1 mmol L^-1^ GA_3_ increased at 5/2 and 15/2°C.

#### Effect of cold stratification on germination of seeds inside silicles

None of the fresh seeds germinated, and after 12 weeks of cold stratification only 21% of them germinated, at 5/2°C (Table 1). A period of dry storage significantly decreased the number of weeks of cold stratification required to break dormancy.

**Table 1:** Effect of cold stratification on germination of 0- and 6-mo-old dry-stored silicles of *Isatis violascens* incubated at 5/2 and 15/2°C in light (mean ± 1 s.e.). Different uppercase letters within a row indicate significant differences between the two temperature regimes and different lowercase letters within a column significant differences among the different treatments.

	Weeks of cold stratification	Final germination (%) of silicles at 5/2°C	Final germination (%) of silicles at 15/2°C
0-months of storage	0	0.0±0.0 ^Aa^	0.0±0.0 ^Aa^
	4	0.0±0.0 ^Aa^	0.0±0.0 ^Aa^
	8	22.0±4.7 ^Bb^	4.0±0.0 ^Aa^
	12	20.6±2.7^Bb^	5.3±0.9^Aa^
6-months of storage	0	1.0±1.0 ^Aa^	0.0±0.0 ^Aa^
	4	56.0±8.6^Bc^	15.0±3.4^Ab^
	8	77.5±6.6^Bd^	42.5±4.3^Ac^
	12*	-*	-*

* Most seeds (98%) germinated during cold stratification at 4°C.

#### Effect of storage in soil in the field on germination and viability

**Fig 5:**
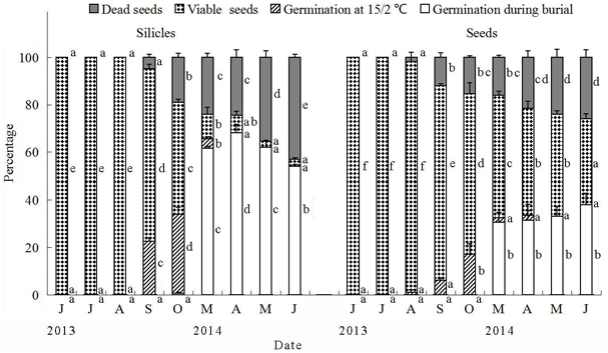
Percentage of germinated, viable and dead seeds in intact silicles and of isolated seeds of *Isatis violascens* retrieved from the soil on year and month indicated and incubated in light at 15/2°C. Portions of bars (seed fates) with different lowercase letters for seeds in silicles or for isolated seeds indicate significant differences in multiple range comparison among retrieval periods. Error bars are + 1 s.e.

In analysis of fates of seeds buried in the field by GLMs with multinomial model (three categories: germinated during burial, nongerminated but viable seeds and dead seeds), the effect of retrieval time (χ^2^ = 269.0, p < 0.001), treatment (intact silicles and isolated seeds) (χ^2^ = 40.3, p < 0.001) and their interaction (χ^2^ = 65.9, p < 0.001) was highly significant. For germination during incubation in Petri dishes, analysis of seed fates by GLMs with a multinomial model (three categories: germinated during incubation, viable seeds and dead seeds) revealed significant effects of retrieval time (χ^2^ = 59.0, p < 0.001). However, there was no significant effect of treatment (χ^2^ = 0.00, p = 0.998) or of the interaction between retrieval time and treatment (χ^2^ = 2.52, p = 0.96). In June 2013, no seeds in silicles or isolated seeds germinated in light at 15/2°C (Fig 5). After 4 mo of burial (i.e. October 2013), 33% and 17% of seeds in silicles and isolated seeds germinated at 15/2°C, respectively. By March 2014 (early spring), mass of silicles, seeds in silicles and isolated seeds had increased by 45.7%, 59.6% and 65.6%, respectively. After 7 mo of burial, 62% of seeds in silicles and 31% of isolated seeds had germinated when bags were exhumed in May 2014. When exhumed in June, 3% of seeds in the silicles (natural dispersal unit) and 36% of isolated seeds were viable.

#### Germination phenology

**Fig 6:**
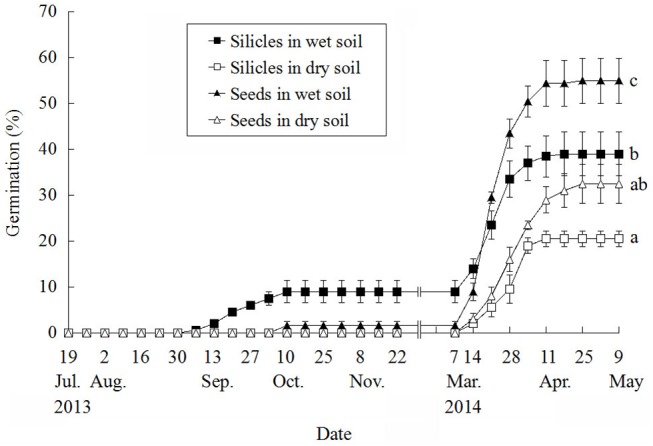
Germination phenology of seeds within silicles and of isolated seeds of *Isatis violascens* on wet (watered) and dry (natural precipitation) soil in the experimental garden. Wet soil means watered, and dry soil means not watered. Significant differences among treatments at the end of the experiment are indicated by different lowercase letters. Bars are ± 1 s.e.

In watered soil, seeds within silicles germinated between 6 September and 11 October 2013, when mean daily maximum and minimum temperatures were 24.5 and 13.5°C, respectively, and isolated seeds germinated between 4 and 11 October, when mean daily maximum and minimum temperatures were 21.6 and 12.1°C, respectively (Fig 6). Total germination in autumn in watered soil was 9% for seeds in silicles and 1.5% for isolated seeds; no germination occurred in nonwatered soil. In spring 2014, most germination of seeds in silicles and of isolated seeds in all treatments occurred from 14 March to 25 April, when mean daily maximum and minimum temperatures were 18.3 and 2.2°C, respectively. Germination percentages of seeds in silicles and of isolated seeds in wet soil were significantly higher than in dry soil (Fig 6). Germination percentage of seeds in silicles in nonwatered soil was significantly lower than that of isolated seeds in wet but not in dry soil. There was no further germination in either wet or dry soil after 25 April 2014.

## Discussion

Our hypothesis that the pericarp of the silicle of *I*. *violascens* inhibits germination was partly supported by data from laboratory studies (Fig 3) and from the germination phenology study in the experimental garden (Fig 6). In the laboratory, some afterripening occurred in seeds inside the silicles and in isolated seeds, as evidenced by 18% and 24% germination, respectively, in darkness at 5/2°C after 9 mo of storage (Fig 3). However, after 12 mo of storage seeds inside silicles germinated to significantly lower percentages than those removed from silicles, indicating an inhibitory effect of the pericarp on germination. In spring 2014, germination percentages of seeds removed from silicles were significantly higher in both watered and nonwatered soil than those of seeds inside silicles, again showing an inhibitory effect of the pericarp on germination.

Since seeds inside silicles imbibed water, the inhibitory effect of the pericarp is not due to water-impermeability of the pericarp or of the seeds. A similar result was found in two other annual Brassicaceae species, namely *Raphanus raphanistrum* [9] and *Diptychocarpus strictus* [10], where neither the fruit nor seed coat prevented water uptake by seeds. Both isolated seeds and those within the pericarp of *D*. *strictus* and of *I*. *violascens* became fully imbibed; however, seeds of *R*. *raphanistrum* within the pericarp did not become fully imbibed.

Germination of seeds incubated in the presence of detached pericarps was not inhibited (Zhou et al., unpublished data), which suggests that the pericarp does not exert its influence by chemical means. Thus, mechanical restriction of the expansion of the embryo appears to be the primary reason for the inhibitory effects of the intact pericarp on germination. After 12 mo of dry storage, seeds inside silicles and those removed from silicles germinated to 38% and 71%, respectively, suggesting that the presence of the pericarp mechanically restricted germination. That is, the embryo in 65% of the seeds inside the silicles did not have enough growth potential to overcome the mechanical restraint of the pericarp. The fact that germination percentages of seeds inside the silicles increased significantly between 9 and 12 mo suggests that as dormancy was broken the growth potential of the embryo increased enough to overcome the mechanical constraint of the pericarp. Another possibility is that the mechanical restraint of the pericarp and/or seed coat became less restrictive to radicle emergence (germination) during afterripening [6]. In any case, dormancy of the embryo (low growth potential) and/or mechanical restriction prevented germination of fresh seeds [9–11,16]. Mekenian and Willemsen [14] and Cheam [15] reported that the low germination percentage of seeds within intact fruits of *Raphanus raphanistrum* was caused by a combination of mechanical resistance of the indehiscent fruit and chemical inhibitors within it. However, Cousens et al. [9] found that removing the seed coat of *R*. *raphanistrum* seeds significantly increased the germination percentage and concluded that the seed coat is more important than the fruit wall (pericarp) in seed dormancy. Ohadi et al. [11] reported that seed dormancy of *Rapistrum rugosum* was caused by the pericarp. However, the pericarp is not the only cause of dormancy in distal seeds of *R*. *rugosum*, which at least in part may be due to the seed coat and/or embryo [26].

Some of our results do not support our hypothesis that the pericarp inhibited germination. In the germination phenology study in autumn 2013, seeds in silicles in watered soil began to germinate earlier, and they germinated to higher percentages than isolated seeds sown in watered soil (Fig 6). Also, in the burial experiment a higher percentage of seed enclosed by the silicles germinated than did isolated seeds. One possible reason for a higher germination percentage of seeds inside the pericarp than of isolated seeds is that the seeds inside the pericarp may have had a higher moisture content. At room temperatures, *I*. *violascens* seeds inside the pericarp required 2–3 h to dry to their pre-imbibition mass (Fig 2). Presence of the pericarp also decreased rate of dehydration of imbibed seeds of *R*. *raphanistrum* [9], *L*. *lehmannii* [16] and *D*. *strictus* [10].

The low germination for both seeds in silicles (9%) and isolated seeds (1.5%) in autumn in wet soil in the experimental garden may mean that only a low percentage of the seeds became nondormant (afterripened) during summer. After 6 mo of dry storage at room conditions, only 11% of the seeds inside the silicles and 24% of isolated seeds germinated in darkness at 5/2°C (optimum condition). In which case, even if the soil is wet during autumn the germination percentage would be low due to seed dormancy. Cold stratification was an effective way to break dormancy in seeds after they had been dry stored for 6 months (Table 1), and after exposure to winter temperature 62% of the seeds inside the buried silicles germinated (Fig 5). Thus, our hypothesis that seeds enclosed by the pericarp would not germinate until the second or some later year after the pericarp had softened, as is the case for the indehiscent silicles of *L*. *lehmannii* [16] and the indehiscent lower siliques of *D*. *strictus* [17], was not supported. The high germination percentage of *I*. *violascens* seeds in the intact silicles in the first spring suggests that few, if any, seeds remain nongerminated until the second or a later spring germination season. Thus, it is unlikely that *I*. *violascens* forms a persistent soil seed bank, i.e. that seeds persist in the soil for more than 1 year (sensu [27]).

The results from the series of experiments conducted to elucidate the role of the pericarp in the germination stage of the life cycle of *I*. *violascens* provide new insight on how timing of germination can be controlled in the temporally unpredicatable (especially with regard to rainfall) cold desert environment. Freshly-matured seeds of *I*. *violascens* were dormant, and little or no germination occurred at any temperature in light or in darkness, even when they were removed from the silicles (Fig 3). Silicles/seeds of *I*. *violascens* are water permeable (Fig 2), and therefore they do not have physical or combinational (physical + physiological) dormancy. Further, the embryo is fully developed (Fig 1); consequently, the seeds do not have morphological or morphophysiological dormancy. Thus, we must conclude that the seeds have physiological dormancy (PD), which is consistent with the kind of dormancy in seeds of other Brassicaceae species [6].

Of the three levels of PD (nondeep, intermediate and deep), nondeep and intermediate were found in *I*. *violascens*. There are three lines of evidence that 20–25% of the seeds had nondeep PD. (1) Some dormancy-break (afterripening) occurred both in seeds in intact silicles and isolated seeds stored dry at room conditions (Fig 3). Similar afterripening in dry storage has been documented in seeds of many species with nondeep PD, and generally the rate of dormancy-break is faster in seeds that become nondormant during summer than in those that become nondormant during winter. For example, in the summer annual *Ambrosia trifida* seeds became nondormant after 3 mo of cold stratification but did so only after 12 mo of dry storage at room temperatures [28]. The rate of afterripening in 20–25% of the seeds of *I*. *violascens* is consistent with the rate of afterripening of seeds that become nondormant relatively quickly when cold stratified. (2) There was an increase in the maximum temperature at which seeds would germinate as dormancy was broken via afterripening, from 5/2°C in fresh seeds to 30/15°C in seeds dry stored for 12 months in the laboratory (Fig 3). This increase in the maximum temperature at which seeds will germinate as dormancy is broken is typical of Type 1 nondeep PD [6]. (3) Promotion of germination of fresh seeds by GA_3_ also is an indication of nondeep PD (Fig 4).

Two observations indicate that the other 75–80% of the *I*. *violascens* seeds had intermediate PD. (1) The effectiveness of cold stratification in breaking dormancy was increased significantly after seeds in silicles were allowed to afterripen in dry storage for 6 mo (Table 1), which is a characteristic of seeds with intermediate PD [6]. (2) GA_3_ was more effective in breaking dormancy after seeds had afterripened for 1 or 12 mo than when they were fresh (Fig 4).

The number of *I*. *violascens* seeds germinating inside silicles in autumn is regulated by (1) the proportion of the seed crop that has nondeep PD and thus can become nondormant by autumn, and (2) adequate rainfall to moisten the soil for several continuous days. The seeds with intermediate PD can not germinate until spring, after they have been cold stratified during winter; seeds need to be imbibed for dormancy-break to occur by cold stratification [6]. Since *I*. *violascens* grows in a cold desert, we might ask how seeds can be imbibed long enough for dormancy break to occur? The answer is that part of dormancy-break occurs in seeds of *I*. *violascens* in the soil during summer, thereby greatly reducing the length of the cold stratification period required to break dormancy. Thus, seeds with intermediate PD are nondormant in spring. The presence of nondeep and intermediate PD in the same seed lot of *I*. *violascens* ensures that (1) some seeds will be nondormant in autumn (Figs 3 and 6) and others nondormant in spring (Fig 5); and (2) even if there is abundant rainfall in autumn, a portion of the seed crop would be prevented from germinating until spring.

Rainfall that is highly variable among seasons and years is a characteristic feature of the cold desert in the Junggar Basin. Depending on the amount and timing of rainfall, seeds of winter annual/ephemeral species may germinate in autumn and/or early spring (mostly) in the Junggar Basin [19,29]. Often, it is too dry in autumn for seeds to germinate. However, when there is sufficient precipitation in autumn seeds of this ecological group of species are stimulated to germinate. Water from snowmelt generally increases water availability in spring, and thus seeds of the winter annuals/ephemerals are more likely to germinate in spring than in autumn. *Isatis violascens* is one member of the cold desert sand dune ephemeral flora whose germination behavior allows its seeds to germinate under natural rainfall in both seasons. In any case, if *I*. *violascens* seedlings that emerge in autumn survive the winter, the plants behave as winter annuals. Plants from spring-germinated seeds behave as spring ephemerals (Lu et al., unpublished data).

Given that *I*. *violascens* is an annual plant reproducing only by seeds and growing in a temporally unpredictable (stochastic) environment, especially with regard to time and amount of rainfall, it is likely that the two levels of PD serve as a bet-hedging strategy [4,30,31]. Such a strategy would increase the geometric mean (the best measure of fitness in a stochastic environment) of the number of offspring across generations [31–33] in the unpredictable environment of the Garbantunggut Desert. Further, although *I*. *violascens* does not form a persistent seed bank (seeds live in soil for ≥ 1 year), it does form a transient seed bank (seeds live in soil for < 1 year) that persists until the spring germination season in the Garbantunggut Desert. Regardless of timing and amount of rainfall in the autumn germination season, the presence of intermediate PD in a portion of the annual seed crop promotes continuation of the population in years in which seeds with nondeep PD germinate in autumn and all the seedlings die due to drought. Thus, the ecological role of seeds of *I*. *violascens* with intermediate PD is to extend the period of persistence of the transient seed bank from autumn to spring. In autumns with sufficient rainfall for germination and survival, individual plants from seeds with nondeep PD would be expected to produce more seeds (and presumably to be more fit) and thus make a higher contribution to population growth than those from spring-germinating seeds with intermediate PD [29]. In short, an advantage to *I*. *violascens* of having two levels of PD is that there is a possibility for some seeds to germinate in the two suitable germination seasons and plants survive and produce seeds.

**Fig 7:**
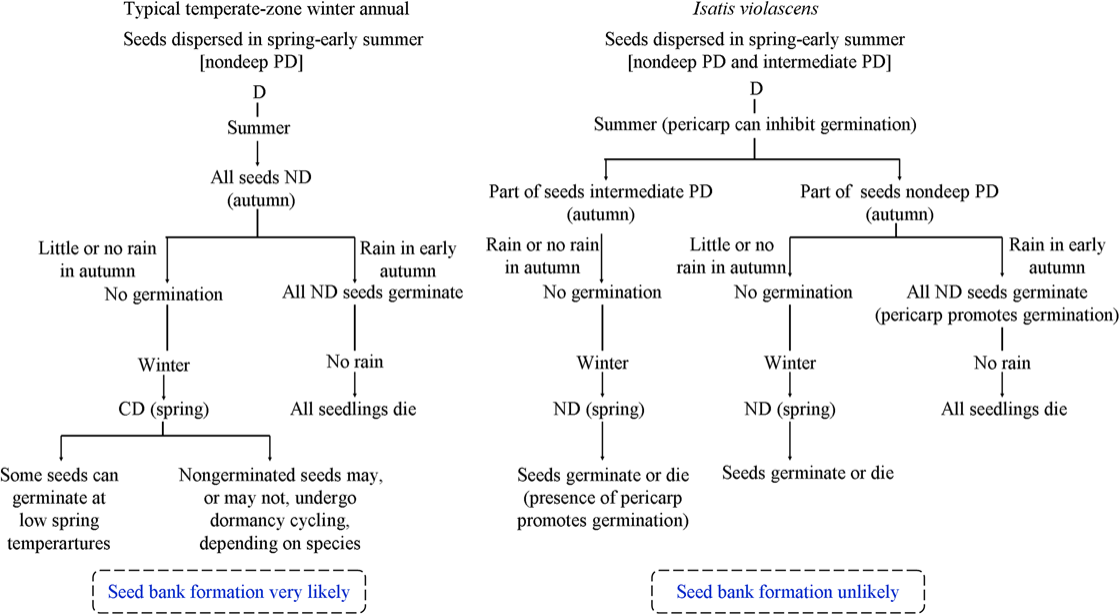
Conceptual model comparing events in the seed stage of the life cycle of a typical temperate-zone disapore-monomorphic facultative winter annual with those of the cold desert facultative winter annual *Isatis violascens*. PD, physiological dormancy; D, dormant; ND, nondormant; CD, conditional dormancy, i.e. seeds can germinate at low but not at high tmperatures.

Many species of temperate-zone facultative winter annuals with water-permeable seeds have been studied, and the seeds had only nondeep PD [6]. In these species, dormancy break occurs during summer, and seeds are nondormant in autumn (Fig 7). The number of seeds that germinates in autumn and percentage of seedling survival depend on amount and timing of rainfall. If seeds fail to germinate in autumn, they lose the ability to germinate at high but not low temperatures, i.e. they enter conditional dormancy and thus can germinate in early spring when temperatures are low. Long term persistence at a population site where the environment is temporally unpredictable may require the formation of a persistent soil seed bank [3], in which the buried seeds may undergo annual dormancy cycling [6]. On the other hand, only 3% of the buried silicles of *I*. *violascens* contained a viable seed after the spring germination season was completed (Fig 5), suggesting that formation of a seed bank is not very important for long-term survival of this species at a population site (Fig 7). The persistence of *I*. *violascens* is promoted by intermediate PD, which prevents germination of a high percentage of a seed cohort in autumn regardless of the amount and timing of rainfall. However, since intermediate PD is broken by cold stratification seeds can germinate in spring, when soil moisture is predictably favorable for seedling survival. Thus, we suggest that intermediate PD in seeds of *I*. *violascens* is playing the same ecological role, in terms of persistence at a site, as the persistent soil seed bank in typical temperate-zone facultative winter annuals.

## References

[1] Al-Shehbaz IA, Beilstein MA, Kellogg EA (2006) Systematics and phylogeny of the Brassicaceae (Cruciferae): an overview. Plant Syst Evol 259: 89–120.

[2] Liu XF, Tan DY (2007) Diaspore characteristics and dispersal strategies of 24 ephemeral species of Brassicaceae in the Junggar Desert of China. J Plant Ecol 31: 1019–1027 (in Chinese with English abstract).

[3] Cohen D (1966) Optimizing reproduction in a randomaly varying environment. J Theoret Biol 12: 119–129. 6015423 10.1016/0022-5193(66)90188-3

[4] Gremer JR, Venable DL (2014) Bet hedging in desert winter annual plants: optimal germination strategies in a variable environment. Ecol Lett 17: 380–387. 10.1111/ele.12241 24393387

[5] Baskin JM, Baskin CC (2004) A classification system for seed dormancy. Seed Sci Res 14: 1–16.

[6] Baskin CC, Baskin JM (2014) Seeds: ecology, biogeography, and evolution of dormancy and germination 2nd edition San Diego: Elsevier/Academic Press.

[7] Nikolaeva MG. (1969) Physiology of deep dormancy in seeds Leningrad, Russia, Izdatel’stvo ‘Nauka’. (Translated from Russian by Z. Shapiro, National Science Foundation, Washington, DC.)

[8] Hu XW, Wang YR, Wu YP (2009) Effects of the pericarp on imbibition, seed germination, and seedling establishment in seeds of *Hedysarum scoparium* Fisch. et Mey. Ecol Res 24: 559–564.

[9] Cousens RD, Young KR, Tadayyon A (2010) The role of the persistent fruit wall in seed water regulation in *Raphanus raphanistrum* (Brassicaceae). Ann Bot 105: 101–108. 10.1093/aob/mcp268 19889801 PMC2794069

[10] Lu JJ, Tan DY, Baskin JM, Baskin CC (2010) Fruit and seed heteromorphism in the cold desert annual ephemeral *Diptychocarpus strictus* (Brassicaceae) and possible adaptive significance. Ann Bot 105: 999–1014. 10.1093/aob/mcq041 20348559 PMC2876001

[11] Ohadi S, Mashhadi HR, Tavakol-Afshari R (2011) Effects of storage and burial on germination responses of encapsulated and naked seeds of turnipweed (*Rapistrum rugosum*) to light. Weed Sci 59: 483–488.

[12] Maxwell CD, Zoble A, Woodfine D (1994) Somatic polymorphism in the achenes of *Tragopogon dubius* . Can J Bot 72: 1282–1288.

[13] Huang ZY, Gutterman Y, Hu ZH, Zhang XS (2001) Seed germination in *Artemisia sphaerocephala*. I. The structure and function of the mucilaginous achene. Acta Phytoecol Sin 25: 22–28 (in Chinese with English abstract).

[14] Mekenian MR, Willemsen RW (1975) Germination characteristics of *Raphanus raphanistrum*. I. Laboratory studies. Bull Torrey Bot Club 102: 243–252.

[15] Cheam AH (1986) Seed production and seed dormancy in wild radish (*Raphanus raphanistrum* L.) and some possibilities for improving control. Weed Res 26: 405–413.

[16] Mamut J, Tan DY, Baskin JM, Baskin CC (2014) Role of trichomes and pericarp in the seed biology of the desert annual *Lachnoloma lehmannii* (Brassicaceae). Ecol Res 29: 33–44.

[17] Lu JJ, Tan DY, Baskin JM, Baskin CC. (2015) Post-release fates of seeds in dehiscent and indehiscent siliques of the diaspore heteromorphic species *Diptychocarpus strictus* (Brassicaceae). Perspect Plant Ecol Evol Syst 17: 255–262.

[18] Wang XQ, Jiang J, Lei JQ, Zhang WM, Qian YB (2003) Distribution of ephemeral plants and their significance in dune stabilization in Gurbantunggut Desert. J. Geograph Sci 13: 323–330.

[19] Wang XQ, Jiang J, Wang YC, Luo WL, Song CW, Chen JJ (2006) Responses of ephemeral plant germination and growth to water and heat conditions in the southern part of the Gurbantunggut Desert. Chinese Sci Bull 51: 110–116

[20] Qian YB, Wu ZN, Zhang LY, Zhao RF, Wang XY, Li YM (2007) Spatial patterns of ephemeral plants in Gurgantünggüt Desert. Chinese Sci Bull 52: 3118–3127.

[21] Li Y, Aladaer Q, Zhang ZF, Zhang YM, Lü GH, Liu B (2014) Phylogenetic relationship and taxonomic status of *Isatis violascens* Bunge (Isatis, Brassicaceae). Acta Bot Boreal-Occident Sin 34: 0902–0907.

[22] Ma SJ, Tan DY (2007) Phenology and sex expression of Junggar desert ephemerals *Neotorularia kovii* and *Isatis violascens* (Brassicaceae). Acta Ecol Sin 27: 0486–0496 (in Chinese with English abstract).

[23] Chen YN, Li Z, Fan YT, Wang HJ, Fang GH (2014) Research progress on the impact of climate change on water resources in the arid region of Northwest China. Acta Geogr Sin 69: 1295–1304 (in Chinese with English abstract).

[24] Sokal RR, Rohlf FJ (1995) Biometry: the principles and practice of statistics in biological research 3rd edition San Francisco: Freeman.

[25] Sun HZ, Lu JJ, Tan DY, Baskin JM, Baskin CC (2009) Dormancy and germination characteristics of the trimorphic achenes *of Garhadiolus papposus* (Asteraceae), an annual ephemeral from the Junggar Desert, China. S Afr J Bot 75: 537–545.

[26] Cousens R, Armas G, Baweja R (1994) Germination of *Rapistrum rugosum* (L.) All. from New South Wales, Australia. Weed Res 34: 127–135.

[27] Thompson K, Grime JP (1979) Seasonal variation in the seed bank of herbaceous species in ten contrasting habitats. J Ecol 67: 893–921.

[28] Davis WE (1930) Primary dormancy, after-ripening, and the development of secondary dormancy in embryos of *Ambrosia trifida* . Amer J Bot 17: 58–76.

[29] Lu JJ, Tan DY, Baskin JM, Baskin CC (2014) Germination season and watering regime, but not seed morph, affect life history traits in a cold desert diaspore-heteromorphic annual. PLoS ONE 9(7): e102018 10.1371/journal.pone.0102018 25013967 PMC4094427

[30] Venable DL (2007) Bet hedging in a guild of desert annuals. Ecology 88: 1086–1090. 17536393 10.1890/06-1495

[31] Simons AM (2011) Modes of response to environmental change and the elusive empirical evidence for bet hedging. Proc R Soc B 278: 1601–1609. 10.1098/rspb.2011.0176 21411456 PMC3081777

[32] Gillespie JH (1977) Natural selection for variances in offspring numbers: a new evolutionary principle. Am Nat 111: 1010–1014.

[33] Philippi T, Seger J (1989) Hedging one’s evolutionary bets, revisited. Trends Ecol Evol 4: 41–44. 10.1016/0169-5347(89)90138-9 21227310

